# Flow in contemporary musicians: Individual differences in flow proneness, anxiety, and emotional intelligence

**DOI:** 10.1371/journal.pone.0265936

**Published:** 2022-03-25

**Authors:** Amy Rakei, Jasmine Tan, Joydeep Bhattacharya

**Affiliations:** 1 Department of Psychology, Goldsmiths, University of London, London, England; 2 Department of Experimental Psychology, University of Oxford, Oxford, England; University College London, UNITED KINGDOM

## Abstract

Flow is a highly focussed state of consciousness that is rewarding, fulfilling, and sought after by many, especially musicians. It is characterised by exceptional levels of concentration, loss of self-consciousness, and competent control over one’s actions. Several personality and non-cognitive traits have been positively linked with flow proneness, such as emotional intelligence; however, anxiety is thought to be the antithesis of flow, yet the relationship between trait anxiety and flow proneness in musicians is not adequately characterised. This study investigated the individual differences in flow proneness in contemporary musicians (*N* = 664), focusing on the interaction of trait anxiety and emotional intelligence. We identified a significant negative correlation between trait anxiety and flow. Emotional intelligence was positively correlated with flow proneness and negatively with trait anxiety. Moderation analysis revealed a difference in the relationship between trait anxiety and flow depending on the level of emotional intelligence; there was no correlation in those with low emotional intelligence, whereas a strong negative relationship was found in those with high emotional intelligence. Finally, hierarchical regression indicated that musical training was the most substantial predictor of all the tested variables and that trait anxiety did not add any predictive power on top of the known predictors. Altogether, this study provided new insights into the possible disruption of flow proneness linked to high anxiety and low emotional intelligence in contemporary musicians.

## Introduction

Flow, named after the effortless flowing of the mind and body [1], is a state of optimal experience that has many similarities to Maslow’s concept of peak experience [2]. Widely considered to be a positive affective state, flow evokes a high level of performance through focused attention [3], competent actions [4], and general feelings of happiness, wellbeing [5], and life satisfaction [6]. Mihaly Csikszentmihalyi, a pioneer in flow research and a founding father of positive psychology, recognised a pattern in the psychological experiences of his subjects while engaged in their favourite activities [7]. Surgeons, rock climbers, chess masters, and artists described feeling totally immersed, motivated by the experience itself, and pursuing the activity for its pure enjoyment. Unsurprisingly, musicians are no exception, finding flow to be critically relevant [8,9]. For example, as flow is highly rewarding and performance during flow is of exceptional nature, musicians may be encouraged to spend thousands of hours mastering their instruments through regularly entering flow state [10]. Flow occurs regularly in musicians [11] though there are wide variations in flow proneness, for example, pianists exhibit lower flow proneness than brass and string players [12] and improvisation, which is relied on more heavily by contemporary musicians, has been critically linked to flow [13]. Flow proneness (or dispositional flow) is the tendency to experience flow states and is correlated with a greater sense of wellbeing in musicians [14]. Therefore, the principal focus of this study is to investigate the individual differences account of flow proneness in contemporary musicians.

Several personality traits have been associated with higher flow proneness in various populations yet anxiety, often considered the antithesis of flow from its very first model [7], has not been fully explored. Musicians are commonly afflicted with anxiety [15]; in fact, research suggests 71% of professional musicians experience high levels of anxiety or panic attacks, and 68.5% have experienced depression [16]. This could be due to external factors like a lack of financial stability [17,18] and stressful working conditions [19], or internal factors such as personality traits, including high levels of neuroticism in professional musicians [20]. Anxiety can be defined as a trait or a state; trait anxiety is centred around the proneness to react in an anxious way to stressful situations [21] and is considered a more stable disposition, whereas state anxiety exists in a moment, in reaction to a specific stimulus [22]. Direct analogies between state and trait anxiety should not be made as they have distinct manifestations [23] and neuroanatomical and functional differences [24]. Specifically, those with high trait anxiety have structural differences in their grey matter, whereas those with high state anxiety have differences in functional connectivity, alluding to the temporary nature of their anxiety. A typical state of anxiety for musicians of all standards is music performance anxiety (MPA) [20], often referred to as stage fright. MPA is well known to be negatively associated with flow [25–27]. Trait anxiety and MPA were shown to be related [28–30]; however, the occupational stressors of elite opera singers, specifically personal resources and personal strain, correlated with trait anxiety more than MPA [23], and MPA is thought to be more closely related to social anxiety than trait anxiety [31], suggesting there are different mechanisms at play.

The relationship between trait anxiety and flow has only been briefly mentioned in two previous studies, but neither focused on musicians. Asakawa found that Japanese college students with lower levels of trait anxiety were more likely to experience flow in daily life [6], while Jackson and colleagues noted that a lack of ability to maintain focus and inappropriate worrying were the cognitive components of anxiety that affected flow states the most [32]. We suggest that trait anxiety would be specifically relevant in the context of flow in musicians due to the high prevalence of anxiety in this population. We predicted that trait anxiety would be negatively related flow proneness in musicians, which would echo findings from MPA studies, but may suggest a more general and stable relationship between trait anxiety and flow.

Another relevant trait in the context of flow in musicians is emotional intelligence (EI) [33]. EI is distinguished into two types: ability EI, which is one’s ability to competently manage, process and utilise emotional information in cognitive tasks [34], and trait EI, which is an affective personality trait, relating to emotion-centric dispositions and self-perceptions [35]. Trait EI is usually measured by psychometric self-reporting, while ability EI is measured by responses to specific task-based scenarios. The stable nature of trait EI is relative to the dispositional nature of trait anxiety and flow proneness and is, therefore, most applicable to this study. Some of the main facets of trait EI include self-esteem, emotion regulation, trait happiness, and stress management [36], all of which have theoretical links to flow proneness; a significant positive relationship between EI and flow proneness has already been identified in musicians [37]. Wrigley and Emmerson [12] found that most music students did not experience a flow state during an examination as a stressful situation, such as this, may increase one’s self-awareness and stress levels rendering them unable to reach flow. Efficient stress management, a feature of trait EI, may significantly improve one’s chances of achieving flow and inevitably produce a more controlled and successful performance. High achievement in music has been associated with increased reports of flow state [38] and given music’s emotional nature, competent musicians are likely to have an increased ability to successfully manage and manipulate musical emotions [39]. Of note, the length of musical training was positively related to trait EI in a sample of student musicians [40].

Furthermore, an interaction between EI and trait anxiety may be present in flow proneness. EI is negatively related to stress and anxiety [41,42] and is positively associated with general feelings of wellbeing [43]; therefore, the negative correlation we have predicted earlier between trait anxiety and flow may have a different degree of intensity at apportioned levels of EI. Specifically, we predict flow proneness to be less related to anxiety in musicians with low EI, as they have reduced control over emotional situations [44], which may negate the expected positive effect of low trait anxiety on flow proneness in this group. Music performance can be an intense emotional experience [45] and if low EI inhibits one’s ability to feel in control in this scenario, it would be contrary to the central flow precondition of ‘sense of control’ [1]. Additionally, individuals with high trait anxiety have an impeded ability to maintain their attention [46], which is a vital component of ‘concentration on the task at hand’, a second central flow precondition [1]. So, while a high EI may offer improved emotional control in those with low anxiety, it may be insufficient to overcome the negative effect of high trait anxiety, implying a more significant relationship between anxiety and flow may be found in this group. We suggest that moderation analysis with EI as the moderator would be the most appropriate way to understand these differences.

Personality traits have been thoroughly researched concerning flow state, and stable associations have been widely agreed upon. For example, flow proneness is thought to be positively associated with dopaminergic-controlled personality dimensions such as stable emotion and positive affect [47], whereas a predisposition to experiencing negative moods, known as neuroticism, may restrict an individual’s opportunity to reach flow [48,49]. Flow states usually occur during autotelic experiences, where the task is intrinsically pleasurable and rewarding [1,49]. Such an experience requires motivation and enjoyment, which may be difficult to achieve if one feels low. Those with high neuroticism may also face barriers such as lower working memory, higher mind-wandering and lower levels of attention control [50,51] which may obstruct one’s access to flow states. An essential feature of flow proneness may be self-regulation [52] which is closely associated with high emotional stability (neuroticism when reverse-scored) and subjective wellbeing [53]. Emotional regulation may be partly genetically determined, expressed as a higher density of dopamine receptors [54] found in flow-prone individuals [47]. It is further thought that emotional regulation is facilitated by EI [33].

Openness-to-experience is highly predictive of flow in both non-musicians [55] and musicians [56] and has been closely related to musical talent and creativity [57,58]. Csikszentmihalyi and Rathunde [59] conjectured that those who are open to challenges or are willing to persist and engage in stimulating activities would be more likely to achieve flow. Furthermore, conscientious individuals have a high level of motivation and are likely to spend time honing skills [48] conducive to reaching the challenge-skill balance, another central pillar of flow [1,60]. Evidence supports this; conscientiousness is the most consistent personality predictor of flow proneness in everyday life [48] and, more specifically, during study time, leisure and while playing an instrument [56]. However, the relations between flow proneness and agreeableness, and extraversion are inconclusive [61–64].

Other than these standard personality traits, several other non-cognitive traits have been associated with flow proneness, and here, we focus only on those appropriate in the context of musicians. The extent to which one feels they have control over events in their life is known as locus of control (LOC), and this may be relevant in the context of ‘sense of control’, the flow dimension [65]. A person with an internal LOC feels responsible for their actions, successes and shortcomings, whereas a person with an external LOC feels their life is controlled by fate or a person of power [66]. It is theorised that an internal LOC directly influences our sense of wellbeing [67] and happiness [68] and is positively associated with flow proneness; in an experimental paradigm using a Tetris computer game, changes to the skills-demands compatibility (challenge-skill balance) greatly affected individuals with a strong internal LOC, whereas those with a weak internal LOC were barely affected [68]. Individuals with a strong internal LOC were more likely to experience flow when they perceived a fit between the challenge of the situation and their skills. Furthermore, individuals with a strong internal LOC tended to experience more control under conditions of skills-demand compatibility, which may be more conducive to flow. Musicians may adjust their skills-demand compatibility by learning suitably difficult music or increasing the demands by performing in front of an audience. Those with a strong internal LOC may be more likely to feel in control while being slightly challenged, making them more susceptible to musical flow. Musical training increases skill level [69] and may also improve the likelihood of finding the challenge-skill balance. However, there is conflicting evidence about the role musical training plays in flow proneness. For example, the duration of the training and the age at which training began were not predictive of flow [37], whereas the broader musical training measure was predictive of flow proneness [70].

A related concept to conscientiousness and training is grit, defined by the level of perseverance one has for their long term goals, even in the face of failure and adversity [71]. Flow theory suggests that skill level needs to be competent to get into flow [9], so individuals with a higher level of perseverance for an activity would develop greater skill and passion, giving grittier individuals a better chance of achieving flow, which is supported by findings in musicians [70]. Similarly, growth mindset is an attitude towards whether one’s abilities are improved through dedication and hard work or are fixed [72]. Although growth mindset is thought to be related to LOC and grit, it was not predictive of flow proneness in musicians [70]. Furthermore, flow may occur regularly in different contexts, and musicians may be more prone to flow in musical scenarios than in daily life. Non-music specialist students and high-achieving and moderately-achieving music students experienced flow regularly during music practice, more than in most other daily activities, except studying [38]. The moderately-achieving music students experienced flow slightly less in music than the other groups (37.2% compared to 50% of the time) and reported slightly more flow in sport. The challenges of underachieving at a specialist music school may impact one’s enjoyment and confidence, thus reducing flow.

Therefore, it is likely that multiple factors influence flow proneness, and understanding how trait anxiety and EI measure next to the variety of established personality and non-cognitive traits would provide critical and novel insight into understanding the individual differences in flow proneness in musicians—the principal aim of this study. Here, we focused exclusively on a large cross-sectional sample of contemporary musicians. Most studies on flow in music have identified correlations between flow proneness and various traits using classical musicians or students; these require corroborating with a broader range of musicians before they can be generalised. It is vital to include contemporary musicians in these discussions as they often have different training experiences, motivation, employment opportunities and commitment to music than their classical counterparts [73].

We hypothesised that features of trait anxiety such as inhibited attention, lack of sense of control, and increased self-consciousness might disrupt flow proneness. We, therefore, predicted that (i) there would be a strong negative correlation between trait anxiety and dispositional flow. Following previous findings on classical musicians [37], we predicted that (ii) EI would be positively correlated with flow proneness. We also hypothesised that (iii) EI would moderate the relationship between anxiety and flow proneness. We theorised that the effect of trait anxiety on flow would hold only when trait EI is high.

Finally, using hierarchical regression, we investigated (iv) whether trait anxiety added any additional predictive power for flow proneness above and beyond these known predictor traits: EI [37], LOC [68], grit [70], conscientiousness, openness-to-experience, and emotional stability [48,55,56], as well as musical training [70].

## Materials and methods

### Design

This non-experimental quantitative research design examines correlational relationships between flow proneness and trait anxiety, EI, LOC, grit, musical training, the Big Five personality traits, daily flow experiences, and flow mindset. Further exploratory correlations compared the nine dimensions of flow against trait anxiety and EI. Moderation analysis explored the relationship between trait anxiety and flow at different levels of EI. Furthermore, hierarchical regression was performed using flow proneness as the dependent variable and the previously associated traits (EI, LOC, grit, conscientiousness, emotional stability, openness-to-experience), musical training and trait anxiety as predictors.

### Participants

The participant total was 664; see Table 1 for a full description. They were identified as contemporary musicians by the main genre they self-selected; all genres were accepted except for classical. In addition, the musicians were asked whether they were classically trained, defined as having “received a number of years of formal training in classical music”; the musical sophistication of the participants was measured using the Goldsmiths Musical Sophistication Index (Gold-MSI) [74]. Data were collected between 15^th^ March and 1^st^ June 2021. The final sample size was substantially higher than the required sample size of 84 that was necessary to identify an effect size of *r =* .30 with 80% power (α = .05, two-tailed) for bivariate correlational analyses [75].

**Table 1:** Participant summary including totals and percentages.

	*N*	%
**Total**	664	
**Age Range**: 18–73	*M* = 26.24	*SD* = 6.93
**Gender**		
Male	469	71%
Female	186	28%
Other	9	1%
**Location**		
United Kingdom	227	34%
United States	86	13%
Germany	48	7%
Australia	44	7%
Italy	25	4%
Netherlands	22	3%
France	22	3%
Canada	19	3%
New Zealand	15	2%
Other	165	24%
**Main Genre**		
R&B/Soul	185	28%
Alternative/Indie	143	22%
Jazz	116	17%
Pop	59	9%
Electronica	42	6%
Other	128	18%
**Main Instrument**		
Guitar	214	32%
Singers	161	24%
Pianist/Keyboard Player	109	16%
Drummers	71	11%
Bass Guitarist/Double Bassists	57	9%
Other	52	8%
**Music Profession**		
Artists	141	21%
Session Musicians	50	8%
Record Producers	43	6%
Singers	40	6%
Other	35	5%
Music Teachers	34	5%
Composers	20	3%
Music Therapists	4	1%
DJs	3	<1%
None	294	44%
**Age Started Music**	*M* = 9.13	*SD* = 3.86
**Musical Sophistication**	*M =* 5.46	*SD* = 0.63
**Classical Training**		
No	391	59%
Yes	273	41%

### Materials

Several questions about the participants’ musical background were presented at the beginning of the study, followed by nine standardised questionnaires. The questionnaires were randomised in blocks according to importance. The first block consisted of flow proneness, trait anxiety, EI, musical sophistication, and personality, considered most significant to the study. Next, the LOC questionnaire was presented, followed by another randomised block including grit and flow in daily life. Flow mindset was the last questionnaire to be presented. In the end, participants were given an option to enter into a prize draw.

Standard demographic data was taken as well as the following questions: “What is your main instrument?”, “What genre of music do you mainly play?”, “Have you been classically trained on any instrument (including voice)? Classically trained essentially means you received a number of years of formal training in classical music.”, “What age did you start to play an instrument (or sing/rap)?”, “Are you a musical professional? If so, what do you do?”. This final question regarding the music profession refers to (i) whether they are a professional musician or not and (ii) the type of profession they were engaged in.

Dispositional flow, or flow proneness, was measured using Jackson and Eklund’s [76] *Dispositional Flow Scale-2* (DFS-2), which includes 36 questions relating to the nine dimensions of flow. It was originally designed for sport but has since been validated for musicians [11,12]. Participants were presented with instructions to draw upon their thoughts and feelings while playing their instrument, whether composing, practising, improvising or performing. The question was intentionally open as musicians may experience flow in any or all these scenarios. Participants were asked to select an answer on a 5-point scale between Never and Always, to statements such as “I am challenged, but I believe my skills will allow me to meet the challenge” and “My attention is focused entirely on what I am doing”.

The trait half of the *State-Trait Anxiety Inventory Y-2* (STAI-T) [22] was used to measure participants’ trait anxiety, i.e. their general tendency to be anxious. There were 20-items measured on a 4-point scale: Almost never to Almost always. The state half of the inventory was not included as most entertainment venues had been closed for 12 months due to the coronavirus pandemic at the time of data collection. Therefore, it was deemed inappropriate as musicians would not have had a recent musical experience to refer to.

*Trait Emotional Intelligence Questionnaire* (TEIQue-SF) [36] measured participants’ global trait emotional intelligence. There were 30 statements such as “I usually find it difficult to regulate my emotions.” and “Generally, I’m able to adapt to new environments.” which were responded to on a 7-point Likert scale (Completely Disagree—Completely Agree).

Musical sophistication was measured using *Goldsmiths Musical Sophistication Index* (Gold-MSI) [74], which consisted of an overall measure and five subscales: active engagement, perceptual abilities, singing abilities, emotional engagement and musical training, the final being the most valid for this study. There were 39 items, and responses were measured on a Likert scale (Completely Disagree—Completely Agree). Mean Gold-MSI subscale and overall scores were used as one question from the perceptual abilities scale (PA_05) was missing from the survey, and therefore it was not possible to use total scores.

*Ten Item Personality Inventory* (TIPI) [77] measured the Big Five personality traits and gave scores for extraversion, agreeableness, conscientiousness, openness-to-experience and emotional stability, which is the reverse-scored equivalent to neuroticism.

*Internality*, *Powerful Others and Chance* (IPC) scale [78] assesses whether participants believe they have control over events in their life or whether they believe things are controlled by people of power or by fate. There were 24 questions that were answered on a 6-point scale. The subscale used in this study was the internal locus of control (LOC), where a high score suggests high internal LOC—the person believes they are in control of their life. The IPC is a multidimensional scale that provides one measure for internal LOC and two separate measures for external LOC: powerful others and chance. An internal LOC has been previously predictive of flow [65], whereas those with external LOC did not enter flow in an experimental setting [68]; therefore, only the internal LOC scale was used in this study.

The *Grit scale* [71] measures individuals’ level of perseverance and passion using 12 items on a 5-point scale. The scale asks respondents to rate how much statements are or are not like them, i.e., “Setbacks don’t discourage me” and “I finish whatever I begin.”

The short version of the *Dispositional Flow Scale* (DFS-2 short*)* [79] was used to assess flow in daily life in general (Flow Daily) rather than focusing on music only. Participants were presented with the following instruction: “Please answer the following questions in relation to your experience of life in general. These questions relate to the thoughts and feelings you may experience during everyday activities.” There were nine questions corresponding to each of the dimensions of flow answered on a 5-point scale between Never and Always.

Finally, a Mindset questionnaire [72] was used to assess attitudes towards motivation and effort in achievement. Those with a growth mindset feel they can improve with effort and those with a fixed mindset feel they cannot change how they are. We were interested in exploring how much participants believed they could increase or decrease their instances of flow: *Flow Mindset*. Participants were presented with a short definition of flow state and how it may be experienced through music, such as being in the zone. They were then asked to indicate how much they agreed with the following sentences.” The three statements were as follows “You cannot really change how much flow you experience.”, “Your tendency to experience flow is something about you that you can’t change very much.”, “Some things or situations can help you get into flow but you cannot really change your basic capacity to experience flow.” These were answered on a 6-point scale from Strongly disagree to Strongly agree.

### Procedure

Data for this study was collected online using the Qualtrics® survey platform. The survey link was distributed across social media platforms such as Twitter, Facebook and Instagram by acquaintances who are professional contemporary musicians and are known to have other musicians as followers. Measures were not adapted for different languages or cultures as the researchers had no intention of reaching participants beyond the UK and US. The participants from further afield were recruited via the same limited number of social posts sent by the professional musicians we used, suggesting they were fans of their music, could understand English-language sufficiently and were also musicians using social media in an analogous way to our target population. The researchers, therefore, assumed that they were culturally similar enough to include in the study. Reliability analysis was run on all variables. Six hundred seventy-three contemporary musicians completed the survey in a mean time of 30.16 minutes after controlling for extremes by removing the longest and shortest 20 durations. Participants were presented with an info sheet and the General Data Protection Regulation (GDPR) form. They were required to agree to share their location data and confirm they were happy to proceed with the study before starting the questionnaire. In the end, participants were given an option to submit their email addresses if they would like to be entered into a prize draw for vouchers. All responses were strictly anonymised before data screening. The study protocol was approved by the Local Ethics Committee of the Psychology Department at Goldsmiths, University of London.

### Statistical analyses

A combination of correlational analyses and regression models was used to analyse relationships between personality dimensions and flow proneness. The open-source statistical package Jamovi for macOS [80] was used for the analysis. Nine multivariate outliers were detected by Mahalanobis Distance analysis and were subsequently removed (*N* = 664); all other assumptions were met. All correlation tests were corrected for multiple comparisons using the false discovery rate [81]. The corrected two-tailed α level was set at .045; bivariate correlations were performed on all main variables and flow dimensions using this adjusted α. Pearson’s *r* correlation coefficients were used as all variables met the appropriate assumptions.

Moderation analysis was run using a general linear model where flow proneness was the dependent variable, trait anxiety was the simple effects variable, and EI was the moderator. Both trait anxiety and EI scales were standardised, and standardised estimates (β) were used where possible, otherwise, unstandardised estimates (B) were employed. Covariates were conditioned using +/- 1 standard deviation. EI and trait anxiety were combined to calculate the interaction term.

Hierarchical linear regression was also performed using flow proneness as the dependent variable, and known predictor variables were entered into blocks according to the prevalence of tested variables in previous flow research [37,48,68,70]. The first block included the three relevant personality traits: conscientiousness, emotional stability, and openness-to-experience. The second block contained musical training, the subscale of the Gold-MSI, which has also been previously predictive of flow. Next, the three non-cognitive traits of interest (LOC, grit and EI) were added. The final block contained trait anxiety.

## Results

Descriptive statistics for all main variables are included in Table 2. The most abnormal skew was observed in the openness-to-experience personality trait (-0.80), but it has been previously identified as a common personality trait in musicians [18]; therefore, it was not considered a concern. Reliability analysis was run on all variables using McDonald’s *ω* [82]. The three main variables (flow, EI and trait anxiety) had high reliability (*ω >*.80), and the majority of the other variables had acceptable reliability (0.82 < *ω* < .57). The low reliability (0.30 < *ω <* .40) for two TIPI variables, openness-to-experiences and agreeableness, were expected [77] due to limited items (= 2) per variable.

**Table 2:** Descriptive statistics of all main variables; flow in music, emotional intelligence, trait anxiety, musical training (subscale 3 of Gold-MSI), Big Five personality traits, grit, locus of control and flow mindset and flow in daily life.

	Mean	SD	Min	Max	*⍵*
Flow	3.59	0.44	2.03	5.00	0.92
EI	4.70	0.62	2.53	6.70	0.82
Trait Anxiety	47.80	10.44	20.00	79.00	0.92
Musical Training	5.07	1.00	1.71	7.00	0.69
Openness-to-experiences	5.89	0.90	2.50	7.00	0.38
Conscientiousness	4.91	1.38	1.00	7.00	0.65
Emotional Stability	4.36	1.48	1.00	7.00	0.71
Agreeableness	4.84	1.09	1.00	7.00	0.33
Extraversion	3.90	1.58	1.00	7.00	0.72
Grit	3.16	0.59	1.67	4.75	0.82
Locus of Control	30.25	5.56	15.00	47.00	0.57
Flow Mindset	4.10	1.31	1.00	7.00	0.81
Flow Daily	3.46	0.47	1.89	5.00	0.75

N = 664; standard deviation (SD), minimum (Min), maximum (Max), ⍵ = McDonald’s omega.

Table 3 shows the bivariate correlation values between our main variables, and Table 4 shows the correlations between the nine dimensions of flow and our main variables. We observed a significant negative relationship between trait anxiety and flow, *r* = -.33, *p* < .001 (Table 3), supporting the first hypothesis. All individual flow dimensions negatively correlated with trait anxiety, except for transformation of time (Table 3); the strongest were loss of self-consciousness, *r* = -.33, *p* < .001, and sense of control, *r* = -.31, *p* < .001. Notably, the negative relationship between trait anxiety and flow was even larger with daily experiences of flow (flow daily), *r* = -.52, *p* < .001. A significant correlation was also observed between flow and EI, *r* = .39, *p* < .001, supporting the second prediction. All flow dimensions were significantly correlated with EI, except for the transformation of time. As previously found, flow was significantly correlated with musical training (*r =* .33, *p* < .001), LOC (*r =* .27, *p <* .001), grit (*r* = .32, *p* < .001), conscientiousness (*r* = .22, *p <* .001), openness-to-experience (*r =* .28, *p <* .001), and emotional stability (*r =* .25, *p* < .001). Weaker but significant associations were also observed with extraversion and agreeableness. Flow daily correlated strongly with the main flow score (which measured flow in musical experiences), *r =* .57, *p <* .001, but there was no relationship between flow and flow mindset, *r =* .00, *p* = .914.

**Table 3:** Pearson’s r correlations between the main variables.

Measure	1	2	3	4	5	6	7	8	9	10	11	12
**1.** Flow	—											
**2.** Trait Anxiety	-0.33***	—										
**3.** EI	0.39***	-0.72***	—									
**4.** Musical Training	0.33***	-0.08*	0.13***	—								
**5.** Extraversion	0.19***	-0.27***	0.34***	0.08*	—							
**6.** Agreeableness	0.13***	-0.16***	0.24***	0.06	0.06	—						
**7.** Conscientiousness	0.22***	-0.35***	0.42***	0.10**	0.07	0.12**	—					
**8.** Emotional Stability	0.25***	-0.70***	0.53***	0.03	0.08	0.19***	0.31***	—				
**9.** Openness to Experiences	0.28***	-0.18***	0.31***	0.15***	0.21***	0.13***	0.07	0.12**	—			
**10.** Locus of Control	0.27***	-0.33***	0.36***	0.07	0.11**	0.11**	0.28***	0.26***	0.19***	—		
**11.** Grit	0.32***	-0.45***	0.49***	0.15***	0.15***	0.16***	0.56***	0.34***	0.21***	0.29***	—	
**12.** Flow Mindset	0.00	-0.17***	0.16***	0.04	0.10**	0.08*	0.07	0.10**	0.07	0.14***	0.11**	—
**13.** Flow Daily	0.57***	-0.52***	0.56***	0.16***	0.25***	0.11**	0.34***	0.39***	0.38***	0.43***	0.46***	0.07

N = 664

*p < .05

**p < .01

***p <. 001 after controlling for FDR.

**Table 4:** Pearson’s r correlations between the 9 flow dimensions and all main variables.

Flow Dimension	Flow	Trait Anxiety	EI	Musical Training	Extra version	Agreeableness	Conscientiousness	Emotional Stability	Openness to Experiences	Locus of Control	Grit
Clear Goals	0.67***	-0.21***	0.30***	0.29***	0.15***	0.07	0.24***	0.12**	0.23***	0.23***	0.34***
Unambiguous Feedback	0.60***	-0.20***	0.28***	0.26***	0.12**	0.11**	0.17***	0.15***	0.11**	0.23***	0.10*
Concentration on Task at Hand	0.69***	-0.24***	0.28***	0.19***	0.10*	0.09*	0.22***	0.16***	0.17***	0.22***	0.27***
Sense of Control	0.75***	-0.31***	0.29***	0.23***	0.10*	0.12**	0.22***	0.23***	0.11**	0.21***	0.25***
Loss of Self-Consciousness	0.59***	-0.33***	0.29***	0.09*	0.08*	0.07	0.07	0.31***	0.20***	0.14***	0.20***
Autotelic Experience	0.63***	-0.22***	0.25***	0.13**	0.16***	0.05	0.07	0.16***	0.21***	0.16***	0.14***
Transformation of Time	0.40***	0.04	0.04	0.07	0.07	0.05	0.01	-0.03	0.13***	0.02	0.06
Merging Action-Awareness	0.68***	-0.15***	0.20***	0.27***	0.13***	0.0*	0.06	0.12**	0.21***	0.16***	0.16***
Challenge-Skill Balance	0.71***	-0.24***	0.28***	0.37***	0.16***	0.10*	0.22***	0.18***	0.21***	0.20***	0.29***

N = 664

* p < .05

** p < .01

*** p < .001 after controlling for FDR.

**Fig 1:**
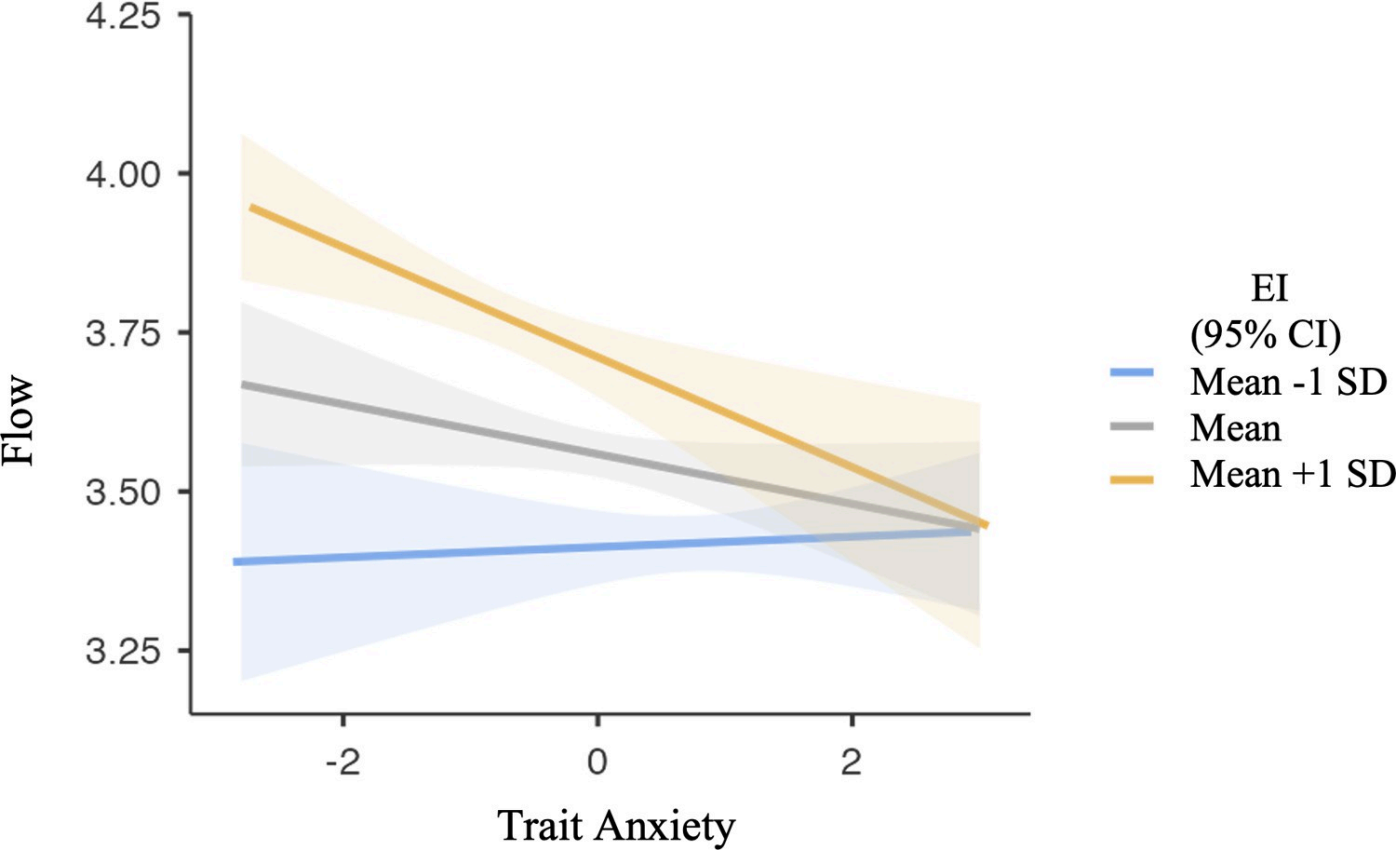
Simple slopes plot showing a significant interaction of EI on the Flow-Trait Anxiety relationship. There is no relationship between Trait Anxiety and Flow in those with low EI, but a significant negative relationship is observed in those with high EI.

A moderation analysis was run to assess whether there was a change in effect at different levels of EI on the relationship between trait anxiety and flow. The outcome variable was flow, the predictor was trait anxiety, and the moderator variable was EI. The interaction term was significant (β = -.11 (-.07, -.02), *t*(660) = -3.65, *p* < .001), (β denotes standardised effect). Simple slopes analysis demonstrated that the flow-anxiety relationship changed at different levels of EI, supporting our third hypothesis. Those with high trait anxiety had low flow proneness at all levels of EI (Fig 1). Additionally, the flow proneness of individuals with low EI is low regardless of their level of anxiety, demonstrated by an insignificant slope, *B* = .01 (-.04, .06), *p =* .756 (*B* denotes unstandardised effect, used only when the standardised statistic was unavailable). High EI was significantly predictive of flow proneness in individuals with low trait anxiety but less so in those with high trait anxiety, generating a significant slope (*B* = -.09 (-.14, -.04), *p* < .001).

In order to examine where trait anxiety stands in comparison to the many previously studied factors influencing flow proneness in musicians, we performed a hierarchical regression with flow proneness as the dependent variable. Table 5 presents the results of hierarchical regression. The total regression model significantly predicted 26% of the variance in flow (*R*^2^ adjusted = .26). The personality traits were collectively able to predict 14% of the variance in flow, *R*^2^ adjusted = .14 (*F*(3, 658) = 36.07, *p <* .001) and musical training predicted an additional 7% variance (*F*(4, 657) = 44.02, *p <* .001). After the non-cognitive traits were added, the model predicted 26% of the variance in flow, *R*^2^ adjusted = .26 (*F*(7, 654) = 33.81, *p <* .001). Trait anxiety was entered last and failed to add any more predictive power to the model (*R*^2^ change = .00, *p* = .363). The largest contribution to the full model was musical training, β = .25 (.18, .31), t(653) = 7.20, *p* < .001. However, EI, (β = .17 (.07, .27), t(653) = 3.23, *p* < .001) and openness to experience (β = .13 (.06, .20), t(653) = 3.70, *p* < .001) were also highly statistically significant. Squared semi-partial correlations demonstrated that musical training uniquely explained 6% (*sr*^2^ = .0625) of the variance, EI explained a small unique variance (*sr*^2^ = .0121), as did openness-to-experience (*sr*^2^ = .0144) and LOC (*sr*^2^ = .0100). All other variables uniquely explained less than 0.5% of the variance, including trait anxiety which only explained 0.09%. The proportion of the variance explained by the model that was not unique to any IV was 21%, suggesting the variables overlapped significantly.

**Table 5:** Hierarchical regression predicting flow proneness including various known predictors and trait anxiety.

			Standardised Beta		
Blocks	Predictor variables	Regression 1	Regression 2	Regression 3	Regression 4
Personality traits	Conscientiousness	0.14***	0.11**	-0.01	-0.01
	Openness to Experience	0.25***	0.21***	0.13***	0.13***
	Emotional Stability	0.17***	0.18***	0.06	0.03
Musical	Musical Training		0.27**	0.25***	0.25***
Non-cognitive and	Grit			0.10*	0.10*
emotional factors	Locus of Control			0.12**	0.11**
	Emotional Intelligence			0.19***	0.17**
Anxiety	Trait Anxiety				-0.05
R2 adjusted		0.14	0.21	0.26	0.26
R2 change			0.07***	0.05***	0.00

Note. N = 680

* p < 0.45

** p < 0.01

*** p < 0.001.

## Discussion

The principal purpose of this study was to examine the individual differences in flow proneness in contemporary musicians. Specifically, we focused on trait anxiety, often overlooked in the studies on flow proneness in musicians, and emotional intelligence (EI), mentioned in a limited amount of flow research. Further individual differences were characterised by personality and non-cognitive traits, including the Big Five, locus of control (LOC), grit, and musical training. The principal findings were as follows: correlation analyses revealed (i) a negative relationship between trait anxiety and flow proneness supporting the first hypothesis, and (ii) EI was positively correlated with flow supporting the second hypothesis. (iii) Moderation analysis revealed that EI significantly interacted with the trait anxiety-flow relationship supporting the third hypothesis. Individuals with low trait anxiety differed in their flow proneness depending on their level of EI; a high EI enabled significantly higher flow proneness if trait anxiety was low, yet trait anxiety was uncorrelated to flow proneness in those with low EI. Positive correlations previously found between flow proneness and LOC, grit, openness-to-experience, conscientiousness and emotional stability were all reflected in our sample. Furthermore, hierarchical regression found that (iv) trait anxiety did not provide any additional explanatory power over and above the known predictors of flow proneness.

The large negative correlation between trait anxiety and flow proneness (*r* = -0.33) found in our sample suggests that the more anxious musicians are, the less likely they will get into a flow state frequently. This is in line with previous studies examining state anxiety, such as music performance anxiety [25–27]. Thus, Csikszentmihalyi’s [7] original suggestion that anxiety is the antithesis of flow appears to be supported, although it may be slightly more nuanced than previously thought, as some flow dimensions were more negatively correlated to trait anxiety than others. In addition, the loss of self-consciousness and sense of control flow dimensions were the most negatively related, suggesting a heightened self-awareness and an insufficient sense of control may be the key barriers for anxious individuals. Of note, our sample of contemporary musicians reported levels of flow proneness comparable to previous studies on musicians [70,83]. However, their trait anxiety (mean of 47.80) was much higher than the reported general population mean of 34.84 [22], offering robust support that musicians suffer from anxiety at a higher rate than the general population [16]. Attitudes towards mental health have changed in recent years [84], which may explain the higher trait anxiety level. However, there may be other factors at play specific to contemporary musicians, such as a dramatic rise in professional competition due to advances in home music technology and the ability to self-release music. Alternatively, higher levels of neuroticism found in professional musicians [17] may account for some of the difference. Practical tools to alleviate the negative impact of anxiety for musicians are desperately needed. While some attempts have been made [85–88], they are mostly plagued by small sample sizes and have not been replicated or expanded upon [89]. One proposed intervention is the use of flow to manage anxiety [12,90]. Our finding of a negative correlation between trait anxiety and flow supports such intervention; however, due to the correlational nature of our study, we cannot establish a causal role of flow in reducing anxiety.

The reported positive relationship between EI and flow proneness (*r* = 0.39) in musicians aligns with a previous study on classical pianists [37] and bolsters foundational research in other domains, such as work [91] and education [92], which suggested an association between flow and EI warrants further exploration. Those with high EI may possess skills such as emotional management and emotional regulation [93], helping with flow proneness, but such coping mechanisms may be insufficient when individuals have high anxiety at clinical levels. Interestingly, our results indicated that highly anxious individuals had equally low flow proneness regardless of their level of EI, whereas a substantial difference was observed in those with low trait anxiety. Trait anxiety and flow proneness were not correlated in those with low EI, but a significant negative correlation was found in those with high EI, which alludes to our prediction that high EI may not negate the negative consequences of anxiety, such as reduced attentional control [46] and an inability to maintain focus [32]. In sum, our moderation analysis indicated the relationship between trait anxiety and flow changes at different levels of EI. It is important to note, however, that this study cannot infer causation, and therefore it is plausible that individuals with naturally low flow proneness may be more prone to anxiety or have lower EI due to fewer flow experiences. Experimental intervention research is needed to establish the direction of this relationship.

EI seems to have a more powerful relationship with flow proneness than trait anxiety, as EI was a significant contributor to the regression model (β = 0.17), yet trait anxiety did not add any explanatory power once all the known predictor variables had been entered (β = -0.05). This could be due to an overarching role of EI encompassing broader emotional regulatory abilities, helping the individual employ a controlled response to emotional situations and a greater capacity to manage stress and anxiety [41,94,95].

Individual differences in flow proneness were also shaped by grit, internal LOC, emotional stability, conscientiousness, and openness-to-experience. The three personality traits were first entered into the regression model as they are considered more stable and are well-researched variables [96], and all were significant predictors. Conscientiousness encompasses similar constructs to grit, such as perseverance [97], and emotional stability and EI have conceptually comparable facets in emotional control [98]; therefore, it is unsurprising that the variance was initially attributed to the personality traits and subsequently moved to the non-cognitive traits. Conclusions should not be drawn from this shift, as it may simply be an artefact from the difference in applied measurement tools. Additionally, the high proportion of shared variance in the main regression model (21%) indicates a significant overlap between the variables. Most of the variables appear to have had some level of collinearity as they had minimal unique contributions.

The tested predictors of flow proneness could explain 26% of the variance in flow, which was significant but not substantial, implying untested variables, such as intrinsic motivation [99] and daily practice hours [70], account for a large proportion of variance. Additionally, evidence from twin studies supporting genetic contributions to flow proneness suggests that the heritability rate is between .29 and .41 [100,101]. There are potential benefits for achieving flow states, such as improved task performance, and Mosing et al. [101] postulated that a portion of the genetic contribution might be non-additive, alluding to the possible influence of natural selection. They also found that flow-prone individuals were less behaviourally inhibited and that these traits and LOC are genetically covariant. Behavioural inhibition is a risk factor for anxiety disorders [102], implying that anxious individuals may be genetically disadvantaged in achieving flow.

Musical training was the most significant unique contributor adding 6% to the full model. Further, its correlation with the challenge-skill balance flow dimension was the largest of all correlations observed between a flow dimension and any other tested variable. Previous findings in musicians had identified the relevance of musical training [25,70], but ours was able to substantiate its importance with a large sample of contemporary musicians. A moderate to high skill level is usually essential for achieving a flow state [1]. Our data support this concept and further suggests that it could be more relevant to flow proneness in musicians than personality and non-cognitive traits.

We measured flow proneness in daily life and found it had a stronger negative correlation with trait anxiety than in musical flow. Music is used in therapeutic situations to help manage stress, anxiety and general negative emotions [103–105]; therefore, musicians may be less affected by their anxiety while playing music. Anxiety may be a more substantial barrier to flow in daily life because the positive effects of music on stress management are not present. This observation raises the possibility of whether flow, particularly in musical scenarios, could be employed as a method to manage anxiety. However, flow in daily life correlated well with flow in musical activities, suggesting some individuals may be simply more flow-prone and given the possible genetic basis for flow [100,101], this could be plausible. Additionally, flow in daily life correlated more substantially with the main personality variables: EI, conscientiousness, openness-to-experience, emotional stability, grit and LOC, suggesting flow in daily life may be more affected by personality, whereas musical flow requires a moderate level of a specific skill, reducing the importance of personality [90]. Alternatively, this difference could have been confounded by using the short version of Jackson and Eklund’s DFS-2 [76], which may be mitigated by using the same full-length measure for both domains in subsequent studies.

There are several limitations to this study that should be considered. First, there was a level of collinearity that was not initially of concern (based on the reasonable VIF and tolerance scores). However, this overlap caused a high percentage of shared variance in the hierarchical regression model and thus made extracting unique variances unfeasible. Principal component analysis may have been helpful to combine some of these variables (conscientiousness with grit or emotional stability with EI and/or trait anxiety) and may have improved the interpretability. However, we did not adopt this analysis as it was important for our study to differentiate the two main variables, EI and trait anxiety. Furthermore, the personality traits were included as they have been the most frequently studied facets of individual differences in flow proneness, and we felt it was essential that they were retained. Second, the low internal reliability found in some of the variables used may have been bolstered if the researchers had run test-retest analysis, which has shown to be more reliable for short scales in other studies [106]. Additionally, the lack of state anxiety data in this study meant that some of the findings could not be compared with previous research on flow in musicians [25–27,90].

However, these findings have provided an exciting foundation for future studies. The relationship between anxiety, EI and flow may change over time. Evidence suggests it is possible to modify trait anxiety [107,108] and therefore flow proneness may improve if the causal pathway is found to be in this direction. Indeed, there are recent suggestions that increasing flow proneness may be an effective tool to reduce MPA [89,90]; therefore, a similar theory could be applied to reducing general levels of anxiety. Equally, managing anxiety through other means such as mindfulness or improving attention regulation [109] may increase flow proneness. In addition, ability EI may also be enhanced [95], which could induce changes in flow, although how this applies to trait EI should be carefully considered. Ultimately, identifying meaningful routes for improving flow proneness through experimental research may have significant beneficial outcomes, such as improving performance and increasing general wellbeing. It may also contribute to mental health research by establishing an effective, inexpensive, and drug-free method to manage anxiety.

In conclusion, this research has identified several individual differences in flow proneness in contemporary musicians, corroborated previous findings with a large cross-sectional dataset and unveiled an essential interaction between trait anxiety and EI. Flow proneness in musical scenarios is partially explained by high EI, openness-to-experience, conscientiousness, emotional stability, internal LOC, grit and musical training. Trait anxiety did not contribute significantly to the hierarchical model; however, moderation analysis indicated that high trait anxiety might limit flow proneness, even in the presence of high EI. Though future research is needed to establish a causal connection between dispositional traits and flow proneness, this study suggests that some contemporary musicians may have an inherent advantage over others in experiencing more flow.
